# Automatic Detection of Small Intestinal Hookworms in Capsule Endoscopy Images Based on a Convolutional Neural Network

**DOI:** 10.1155/2021/5682288

**Published:** 2021-11-24

**Authors:** Tao Gan, Yulin Yang, Shuaicheng Liu, Bing Zeng, Jinlin Yang, Kai Deng, Junchao Wu, Li Yang

**Affiliations:** ^1^Department of Gastroenterology and Hepatology, West China Hospital, Sichuan University, Chengdu, 610041 Sichuan, China; ^2^School of Information and Communication Engineering, University of Electronic Science and Technology of China, Chengdu, 611731 Sichuan, China

## Abstract

Ancylostomiasis is a fairly common small bowel parasite disease identified by capsule endoscopy (CE) for which a computer-aided clinical detection method has not been established. We sought to develop an artificial intelligence system with a convolutional neural network (CNN) to automatically detect hookworms in CE images. We trained a deep CNN system based on a YOLO-V4 (You Look Only Once-Version4) detector using 11236 CE images of hookworms. We assessed its performance by calculating the area under the receiver operating characteristic curve and its sensitivity, specificity, and accuracy using an independent test set of 10,529 small-bowel images including 531 images of hookworms. The trained CNN system required 403 seconds to evaluate 10,529 test images. The area under the curve for the detection of hookworms was 0.972 (95% confidence interval (CI), 0.967-0.978). The sensitivity, specificity, and accuracy of the CNN system were 92.2%, 91.1%, and 91.2%, respectively, at a probability score cut-off of 0.485. We developed and validated a CNN-based system for detecting hookworms in CE images. By combining this high-accuracy, high-speed, and oversight-preventing system with other CNN systems, we hope it will become an important supplement for detecting intestinal abnormalities in CE images. This trial is registered with ChiCTR2000034546 (a clinical research of artificial-intelligence-aided diagnosis for hookworms in small intestine by capsule endoscope images).

## 1. Introduction

Remarkable progression in the investigation and diagnosis of small bowel lesions, such as tumors, ulcerations, enteritis, and parasites, by capsule endoscopy (CE) has been made in recent years [1–4]. An innovative endoscopic capsule passes through the GI tract, capturing approximately 40,000-60,000 images per patient. A heavy burden is imposed on physicians to screen lesions from a massive number of images; as especially when lesions are present only in several frames, they may easily be missed by the physicians due to fatigue or oversight. In order to reduce the burden on physicians and improve the efficiency and accuracy of endoscopic diagnosis, computer software technology has begun to be applied to this field. With the continuous development of the combination of computer software technology and endoscopic diagnosis [5], many computer-aided methods have been formed, and such methods are promising for the detection of many small intestinal abnormalities [6–8], such as bleeding [9], erosions [1], ulcerations [1], angioectasias [10], and protruding lesions [11], such as polyps, nodules, epithelial tumors, stromal tumors, and venous structures.

Ancylostomiasis in the small intestine is still a fairly common small bowel parasite disease in some regions of developing countries, in addition to the southern part of China, and is one of the etiologies of obscure gastrointestinal bleeding (OGIB). Patients see a doctor due to unknown chronic hypoferric anemia or positive occult blood test results, and the diagnosis can be established by CE [12]. In our earlier report on automatic detection software based on the color and morphological features of hookworms [13], the ability of the software to detect hookworms was poorer than its ability to detect the lesions mentioned above due to algorithm imperfections [13].

Recent reports have shown that convolutional neural networks (CNNs), a new type of “deep learning” algorithm in the artificial intelligence (AI) field, have succeeded in detecting many lesions in medical images, such as pulmonary nodules [14, 15], breast lesions [16, 17], skin cancer [18], and early gastrointestinal cancers [19, 20], in addition to the ones mentioned above in CE images. In particular, the reason for the popularity of CNNs lies in their ability to extract the characteristics of images based on accumulated images, which makes them useful for analysis of medical images and for image-based detection. Once the detection module has been obtained, such network scan automatically and rapidly process large numbers of images.

Although many promising diagnostic results have been obtained from CE images using CNNs, there have been few analyses of the detailed classification of parasites, including hookworms, according to color and morphology, and there have been few related clinical reports in the field.

In this study, we developed and validated a CNN-based system for the automatic detection of hookworms in small bowel CE images. We used 11236 CE images for training and 10,529 independent CE images for testing.

## 2. Materials and Methods

### 2.1. Preparation of the Training Image Set

**Figure 1:**
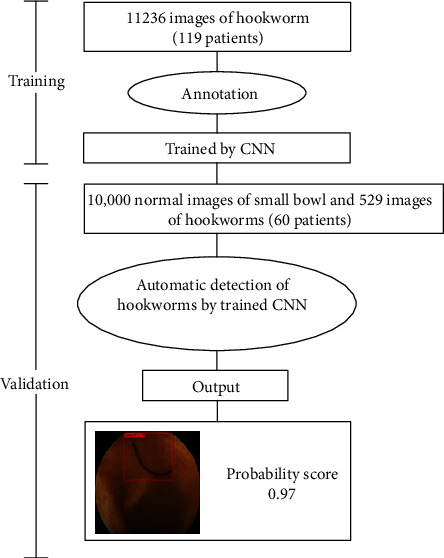
The flowchart of this study.

The study design was reviewed and approved by the Ethics Committee of West China Hospital, Sichuan University (No.2020 (290)), and it was registered in the Chinese Clinical Trial Registry (No. ChiCTR2000034546) on July 9th, 2020. This was a retrospective study using anonymized CE images, and informed consent was waived for patients included in the study. CE images taken between May 2007 and December 2020 were obtained from a single institute (The West China Hospital, China). All of the CE examinations for our study were performed using an OMOM CE device (Jinshan Technology CO., Chongqing, China). The CE findings obtained by 3 endoscopists were recorded prospectively in an electronic database. As a training image dataset for the CNN system, we collected 11236 images of small bowel hookworms from 119 patients between May 2007 and August 2016; the flowchart of this study is listed in Figure 1.

### 2.2. Preparation of the Validation Image Set

A total of 10,529 independent CE images from 60 patients obtained between November 2016 and December 2020 were prepared as a validation image set. Of these CE images, 529 showed hookworms in the small bowel, and 10,000 images showed a normal small bowel mucosa (Figure 1).

### 2.3. CNN Algorithm

To construct an AI-based diagnostic system, we used a deep detection neural network called the YOLO-V4 (You Look Only Once-Version4) as the main part and a small classification neural network as the supplementary part. YOLO-V4 is a deep CNN that consists of 53 or more layers, and the classification network consists of 3 layers [21, 22]. All regions showing hookworms in the training set were manually annotated for this study by 2 expert endoscopists (Tao G. and Jinlin Y.). The annotation was performed separately, and consensus was later determined. These images were fed into the YOLO-V4 and classification networks through two frame works, Pytorch and TensorFlow. The diagnostic system was taught to recognize the areas within the bounding boxes as hookworms and the other areas as background.

All layers of the YOLO-V4 and classification network were fine-tuned using Adam (adaptive moment estimation) gradient descent. Each image was resized to 416 × 416 pixels in YOLO-V4 and 256 × 240 in the classification network; the bounding box was also resized accordingly. These values were determined by trial and error to ensure that all data were compatible with the system.

### 2.4. Outcome Measures and Statistics

The primary outcomes included the area under the receiver operating characteristic curve (ROC), sensitivity, specificity, and accuracy of the CNN for detecting hookworms. First, 2 expert endoscopists (Tao G. and Jinlin Y.) manually annotated all hookworms with green rectangular bounding boxes in the validation set (“true boxes”) used for this study. The annotation was performed separately, and consensus was later decided. Finally, the annotations of 11236 images with hookworms were modified at the stage of consensus. The trained CNN system marked the region of hookworms with red rectangular bounding boxes (“CNN boxes”) in the validation set and provided a hookworm probability score (range, 0 ~ 1). The higher the probability score was, the greater the confidence that a region identified by the CNN contained hookworms.

The CNN-based detection system was validated by evaluating its ability to distinguish whether each image contained hookworms. The following definitions were used: (1) If the image contained one or more hookworms, and the CNN box had a probability score larger than the cut-off value on one or more hookworms, then it was considered a true positive, while it was considered a false negative if there were no CNN boxes with a probability score above the cut-off value. For an image without a hookworm, if no CNN boxes had a probability score larger than the cut-off value, then this image was counted as a true negative, and it was considered a false positive if there was a CNN box with a probability score larger than the cut-off value. (2) When the overlapping area between the CNN box and the true box covered more than 70%, the CNN box was defined as correct.

Comparisons were performed by univariate analysis using the Pearson chi-squared test. A *P* value < 0.05 was considered statistically significant. The receiver operating characteristic (ROC) curve was plotted by varying the threshold of the probability score, and the area under the curve (AUC) was calculated to evaluate the system's detection ability. The sensitivity, specificity, and accuracy of the CNN in detecting hookworms were calculated using cut-off values for the probability score according to the Youden index [23, 24].

The data were analyzed statistically using the SPSS software (version 17).

## 3. Results

### 3.1. Capability of the CNN in Detecting Hookworms

The characteristics of the patients in the training and validation datasets are shown in Table 1. The most common cause of hookworm infection was touching soil containing filariform larvae of hookworms with bare hands, feet, or other parts of the body or consuming food containing filariform larvae of hookworms, but in both datasets, the patients could not provide the relevant information of history of infection due to chronic and occult incidence. The validation set consisted of 10,529 images from 60 patients (male, 43.3%; mean age, 59.7 ± 11.9 years). The trained CNN required 403 seconds to evaluate the images, with an average speed of 26 images per second.

**Figure 2:**
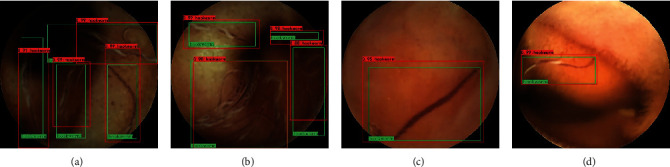
Representative images of multiple hookworms and single hookworm correctly detected by the convolutional neural network (CNN) in the validation set (green box, true lesion; red box, region identified as hookworms by the CNN).

**Figure 3:**
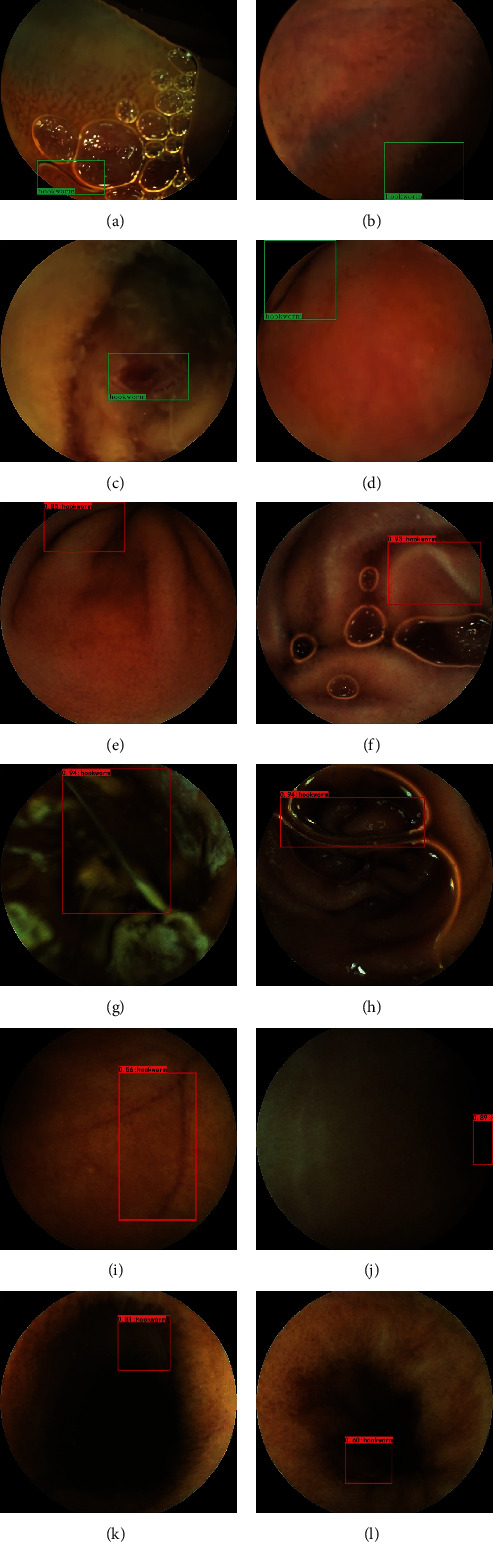
Representative images classified differently by experts and the CNN in the validation set (green box, region identified as a hookworm by experts; red box, region identified as a hookworm by the convolutional neural network (CNN); number, the probability score of the CNN reading). Samples of false-negative images due to (a) bubble, (b) darkness, (c) debris, and (d) vascular shadow. Samples of false-positive images due to (e) fold, (f) fold, (g) debris, (h) bubble, (i) vascular shadow, and (j) darkness. (k, l) True hookworm detected by the CNN that were missed by the experts.

Figures 2(a)–2(d) show representative regions correctly marked by the CNN, and Figures 3(a)–3(l) show typical regions classified differently by the experts and the CNN. As shown in Table 2, false negative images (*n* = 56) were classified into 4 categories based on the cause of the false negative read: poor demarcation mainly caused by the debris and darkness (Figures 3(b) and 3(c)), similarity to the edge of a bubble (Figure 3(a)), similarity to a submucosal vascular shadow (Figure 3(d)) and smallness. On the other hand, false positive images (*n* = 895) were classified into 6 categories based on the reason for the false positive read: darkness (Figure 3(j)), a bubble (Figure 3(h)), debris (Figure 3(g)), vascular shadow (Figure 3(i)), a fold (Figures 3(e) and 3(f)), and smallness. Two true hookworms missed by the experts were detected by the CNN (Figures 3(k) and 3(l)).

**Figure 4:**
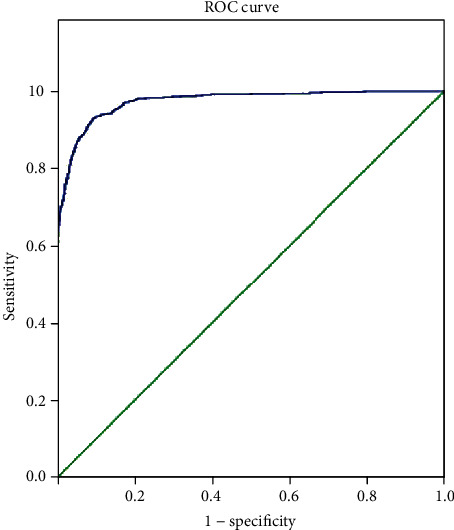
The receiver operating characteristic curve of the convolutional neural network for detecting hookworms. AUC: area under the curve; CI: confidence interval.

The AUC of the CNN used for detecting hookworms was 0.972 (95% confidence interval (CI), 0.967-0.978; Figure 4).

According to the Youden index, the optimal cut-off value for the probability score was 0.485; therefore, regions with a probability score of ≥0.485 were recognized as containing hookworms by the CNN. At this cut-off value, the sensitivity, specificity, and accuracy of the CNN were 92.2%, 91.1%, and 91.2%, respectively (Table 3).

Table 4 shows the changes in sensitivity, specificity, and accuracy when the cut-off value for the probability score was increased in 0.1 increments from 0.2 to 0.9.

At a cut-off value of 0.485, in 529 images, 838 hookworms in 473 images were detected by 641 CNN “true boxes,” 20 hookworms in 12 images were detected by CNN “true boxes,” and 32 hookworms in 12 images and 55 hookworms in 44 other images were not detected by the CNN. Among10000 images, two hookworms were detected in two images by 2 CNN “true boxes” but not by the expert endoscopists.

The detection rate of the CNN for different types of infection was as follows: there was no difference in detection rate for single hookworms and multiple hookworms (88.8% vs. 92.0%, *P* = 0.11) or between fully blood-fed hookworms and partly blood-fed hookworms (89.7% vs. 91.0%, *P* = 0.59). When the CNN software used in this trial was used to detect hookworms once again by using the same images of validation dataset to appraise the reproducibility, the results were completely consistent with those of the previous test.

## 4. Discussion

We developed a CNN-based system for automatic detection of hookworms in small bowel CE images. The trained CNN was shown to detect hookworms in independent test images with a high accuracy of 91.2% (AUC, 0.972). Moreover, the results were equally good for single hookworms and multiple hookworms and for hookworms that were partly or fully blood-fed. The detection process of the CNN software system was not random.

For the evaluation of small bowel mucosal damage, we speculated that it is essential to consider intestinal parasites as well as other intestinal lesions. A previous work reported the automatic detection of hookworms based on an edge extraction network and classification network in 2016; however, although the preliminary results confirmed the ability of CNN to detect hookworms, consistent with other literature findings [2], fewer images of hookworms were used, limiting the ability further clinically validate the findings [25]. Since 2015, some studies have reported the effectiveness of the deep learning-based analysis of CE images for identifying intestinal lesions such as angioectasia, ulceration, erosion, polyps, hemorrhages, and protruding masses [9, 11]. However, there have been no clinical studies or reports on intestinal parasites such as hookworms, roundworms, and tapeworms. One reason is that enough samples had not been obtained. In this study, the accumulation of case images allowed us to further clinically validate an automatic detection system for hookworms in CE images using a deep learning method. Using more than 11000 training images, our CNN was able to achieve “self-learning” and attain a high level of detection (AUC, 0.972). Interestingly, the CNN detected 2 true hookworms that the experts had missed. The experts likely missed these lesions because the surrounding dark background caused by coffee-like blood was a similar color as the bodies of fully blood-fed hookworms. It is surprising that the CNN system revised our oversight during the course of a high-speed review at more than 26 images per second. There are still some inadequacies in the CNN system that should be improved in our future work. More than half of the classification errors made by the CNN, regardless of whether they were a false positives or false negatives, were mainly caused by three interference factors: darkness, debris, and bubbles. Darkness often resulted from the coffee-like blood due to the effect of acid and bacterial decomposition after blood oozes from the wounds in the intestinal wall caused by massive hookworms. In addition to the CNN detection method mentioned above, image light enhancement technology is the next strategy we should use. In contrast to darkness, some debris and bubbles have similar morphologies as the bodies of hookworms (see Figures 3(g)–3(i)), which have folds and submucosal vascularity; thus, a sufficiently large number of images with these findings will be used to train the CNN system to improve its specificity in detecting hookworms. Obviously, the sensitivity of detection may be negatively influenced by insufficient lighting, poor bowel preparation, and other factors such as poor focus and a small expose of hookworm. In addition, bubbles, debris, bile, etc. were found to affect the sensitivity when present with hookworms (see Figures 3(a) and 3(c)). The CNN system had difficulty detecting hookworms, possibly because it was confused by bubbles, debris, bile, etc. This result suggests that when bowel cleansing is poor, the sensitivity of the detected lesions can be improved if the CNN system learned those findings [26].

Moreover, the results showed that the sensitivity, specificity, and accuracy of the CNN were 92.2%, 91.1%, and 91.2%, respectively. We recommend that the goal of the CNN system be to maintain an auxiliary diagnosis in clinical practice until it is supported by large-scale effective results with high sensitivity. Although we used the Youden index as the standard cut-off value in this study, we should improve the capabilities of this system and search for the best cut-off value with higher sensitivity during further clinical validation.

Other future works include applying this method to other parasites mentioned above to extend the utility of the CNN system. In clinical practice, this detection system should be combined with other CNN detection systems for intestinal abnormalities [3] such as those for detecting protruding lesions [11], erosions and ulcerations [1], enteritis, intraluminal hemorrhage [9], and angioectasia [10], and the clinical effects should be further evaluated.

Our study had several limitations. First, this was a retrospective study, although as many samples as possible were obtained from one medical unit. Second, our detection system should be validated in other hospitals by using multicenter data. Third, our CNN system was developed and investigated by using images from the OMOM CE system, and it is unclear whether images from other CE systems can be used with this detection system.

In conclusion, we developed and validated a CNN-based detection system for hookworms in CE images. We hope this high-accuracy, high-speed, and oversight-preventing system will become important for detecting intestinal abnormalities in CE images in combination with other CNN detection systems.

## Figures and Tables

**Table 1:** Characteristics of patients in the training and validation datasets.

Characteristics, *n* (%)	Training dataset (*n* = 119)	Validation dataset	
		Hookworms (*n* = 40)	Normal (*n* = 20)
No. of images	11236	469	10000
Age (years), mean (SD)	56.2 ± 14.8	63.7 ± 9.0	52.3 ± 12.6
Sex, male	59 (49.6)	17 (42.5)	9 (45.0)
Exam indication for CE			
OGIB	94	28	6
Abdominal pain	23	8	14
Anemia	22	7	2
Abnormal results	8	3	1
Abdominal distention	3	1	3
Diarrhea	3	0	1
Screening	3	0	0
Constipation	1	3	0
No. of hookworms			
≤3	46	14	
>3	73	26	
Location of hookworm			
Jejunum	69	33	
Ileum	13	1	
Diffuse	37	6	
Eosinophile granulocyte	3	3	
Hookworm ovum^∗^	3	1	
Concomitant lesions			
No other lesions	46	16	7
Enteritis	26	8	5
Polyp	19	3	6
Submucosal mass	18	12	4
Angioectasia	13	2	3
Erosion/ulcer	9	7	3
Miscellaneous^	8	3	4

Values are number (%) except where indicated otherwise. SD: standard deviation; CE: wireless capsule endoscopy; OGIB: obscure gastrointestinal bleeding. ^∗^Hookworm ovum of stool routine. ^The causes of miscellaneous cases included lymphatic dilatation (*n* = 2), diverticulum (*n* = 1), roundworm (*n* = 2), intestinal scar (*n* = 2), and stromal tumor (*n* = 1) in training dataset and lymphatic dilatation (*n* = 3), intestinal scar (*n* = 1), vein tumor (n = 1), and stromal tumor (*n* = 2) in validation dataset.

**Table 2:** Causes of discrepancies in classifications by the experts and by the CNN.

	*n* (%)
False-negative lesions (*n* = 56)	
Poorly demarcated	(53.6)
Debris	12 (21.4)
Darkness^∗^	17 (30.4)
Poor focus	1 (1.8)
Similarity to the edge of the bubble	7 (12.5)
Similarity to the submucosal vascular shadow	17 (30.4)
Smallness	2 (3.6)
False-positive lesions (*n* = 895)	
Darkness	32 (3.6)
Bubble	179 (20)
Debris	516 (57.7)
Vascular shadow	73 (8.2)
Smallness	3 (0.2)
Fold	92 (10.3)
Hookworms overlooked by experts (*n* = 2)	
True hookworms	2 (100)

^∗^Caused by dark view or coffee-like bloody fluid.

**Table 3:** Classification of images predicted by the CNN technology.

CNN classification	Classification of endoscopists		Total
	Hookworms	Normal mucosa	
Hookworms	659	897	1556
Normal mucosa	56	9178	9234
Total	715	10075	10790

Sensitivity, 92.2%; specificity, 91.1%; accuracy, 91.2%.

**Table 4:** The ability of classification for each cut-off value.

Cut-off value (*P* value)	Sensitivity (%)	Specificity (%)	Accuracy (%)
0.1	98.7	65.3	67.5
0.2	98.2	77.5	78.9
0.3	95.5	84.0	84.8
0.4	94.0	88.3	88.7
0.485^∗^	92.2	91.1	91.2
0.5	91.6	91.5	91.5
0.6	87.3	94.4	93.9
0.7	81.4	96.4	95.4
0.8	72.4	98.2	96.5
0.9	61.7	99.5	97.0

^∗^Calculated according to the Youden index.

## Data Availability

Data are available on request through the authors themselves (contact: gantao@wchscu.cn).

## Abbreviations

AI: Artificial intelligence
AUC: Area under the curve
CI: Confidence interval
CNN: Convolutional neural network
CE: Capsule endoscopy
Fig: Figure.
